# Crouzon syndrome complicated with binocular strabismus and extraocular muscle fibrosis: a case report

**DOI:** 10.1186/s13256-022-03709-9

**Published:** 2023-02-09

**Authors:** Yuling Niu, Jin Xu, Rushan Ye, Zixian Dai, Ling Jin, Wenwen Geng

**Affiliations:** Department of Ophthalmology, People’s Hospital of Shenzhen Baoan District, Shenzhen, 518101 China

**Keywords:** Strabismus, Crouzon syndrome, Extraocular muscle fibrosis, FGFR2, Genetic, Shallow orbits

## Abstract

**Background:**

Crouzon syndrome, a rare genetic disorder characterized by premature closure of coronal sutures, results in skull and facial deformities along with abnormal brain and ocular development.

**Case presentation:**

Here, we report a case of a 27-year-old ethnic han male patient who presented with complex binocular strabismus secondary to Crouzon syndrome. At the time of surgery, extraocular muscles were found to be fibrotic and results of the pathological examination revealed degeneration of muscle fibers, which were replaced by adipose tissue. The entire exome sequencing DNA testing indicated that the patient and his father possessed the fibroblast growth factor receptor 2 (*FGFR2*) gene c.G812T:p.G271V heterozygous mutation. Binocular strabismus corrective surgery was performed in this patient with a satisfactory outcome.

**Conclusions:**

This case demonstrates that Crouzon syndrome patients can show an *FGFR2* gene c.G812T:p.G271V mutation and display clinical symptoms such as extraocular muscle fibrosis, exotropia, exophthalmos, and a pointed head deformity.

## Introduction

Crouzon syndrome, also known as Virchow syndrome, is a rare genetic disorder characterized by premature closure of cranial sutures [1]. This condition results in skull and facial deformities including a pointed head, parrot-like nose, exophthalmos, shallow orbits, and exotropia. The disease is generally inherited in an autosomal dominant manner [2] and has an incidence of 1/60,000 to 1.65/100,000 [3]. The mutant gene in most patients is located on the fibroblast growth factor receptor 2 (*FGFR2*) gene fragment on chromosome 10q25-q26 [4–6]. Common eye damage resulting from the premature closure of cranial sutures due to Crouzon syndrome is attributable to increased intracranial pressure, nerve traction, and/or narrowing of the optic foramen, all of which can contribute to optic nerve atrophy and even blindness [7]. The pressure exerted on the orbit produces a shallowed orbital cavity, enlarged angle of the outer wall, exophthalmos, a tortuous route of the optic nerve, and abnormal shapes and positions of extraocular muscles [8]. Crouzon syndrome is often accompanied with strabismus, with general exotropia accounting for 46.7% and esotropia accounting for 20.0%. In particular, the V-pattern is the most commonly reported strabismus associated with this condition [9]. No obvious differences in muscle and collagen fibers are observed in patients with Crouzon syndrome as compared with healthy individuals [10]. Coats *et al*. 2000 reported that in 7 of 9 patients with Crouzon syndrome and V-strabismus, the superior oblique muscle was absent [11]. Most Crouzon syndrome patients with strabismus require strabismus surgery. If severe craniofacial bone deformities are combined with strabismus, it is recommended that craniofacial plastic surgery be performed in infancy prior to strabismus surgery, with correction of eye position being the main goal of this surgery.

## Patient information

A 27-year-old ethnic han male patient arrived at our hospital in March 2021 presenting with a bilateral deviation in eye position that had been present since birth. The patient had a pointed head, protruding eyes, and normal mental development. There was a normal occlusion of the patient’s teeth, and fingers/toes were not deformed. While the patient’s grandparents, mother, and sisters exhibited a normal appearance, his father displayed similar symptoms to the patient.

### Clinical findings

**Fig. 1:**
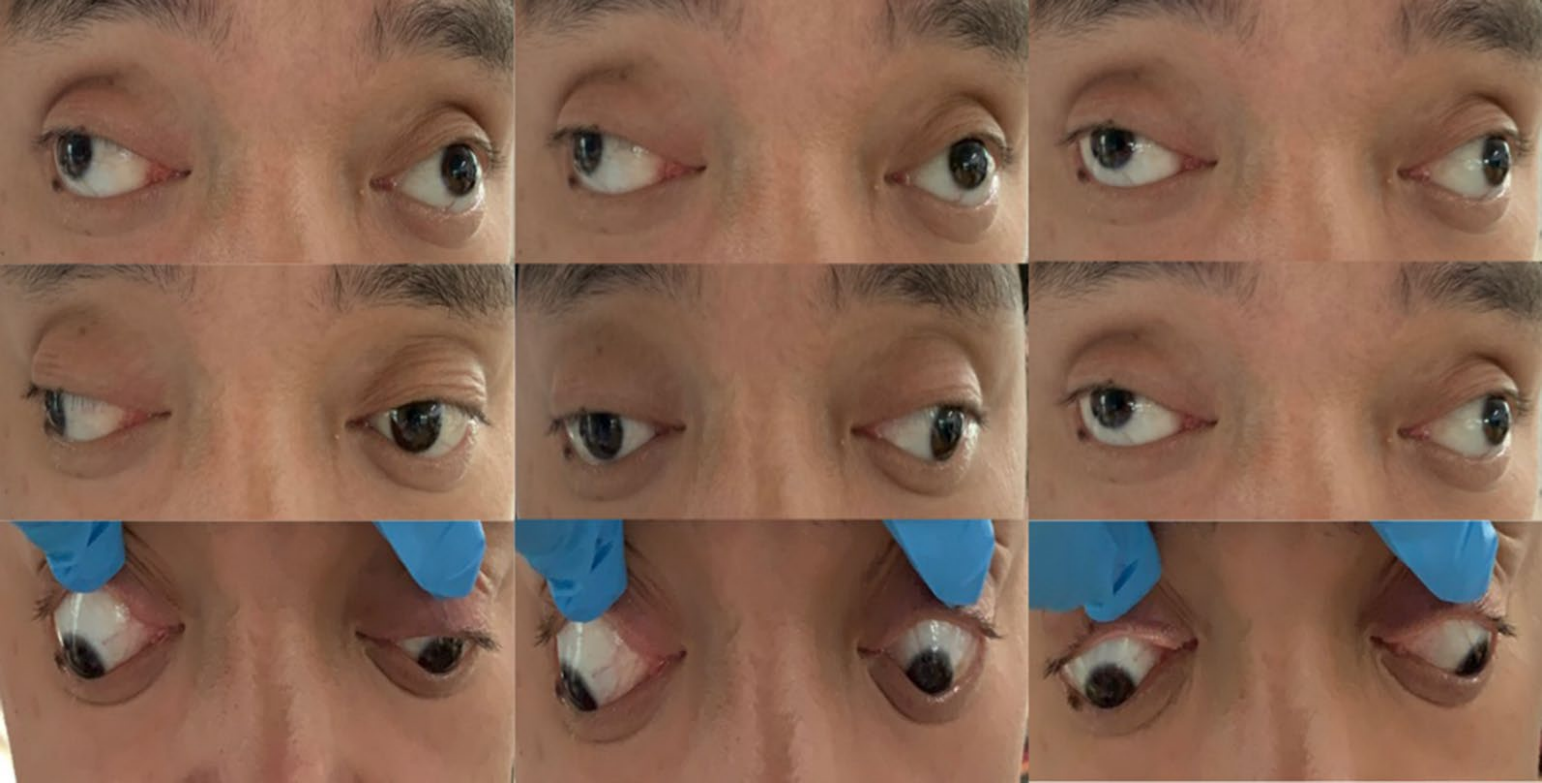
Eye positions prior to surgery as shown in nine directions of gaze. Internal rotations of both eyes were decreased by 2–3 mm

Examination results revealed that his visual acuity was 20/25 (−0.75/−0.75 × 143 20/20) in the right eye and 20/20 in the left eye. Non-contact tonometry test results indicated that the intraocular pressure was 18 mmHg in the right eye and 19 mmHg in the left eye and the amount of ocular protrusion was 28 mm in both eyes. No significant abnormal changes were observed in anterior and posterior segment exams in both eyes, and Hirschberg test results showed that the corneal reflex was ≥ 45°. The prism alternate cover test (PACT) revealed a constant exotropia (XT) of 100∆ at near and distance fixation, 100∆ XT at upward gaze, and 90∆ XT at downward gaze. No titmus was observed. Eye movements were normal, although internal rotation was slightly inadequate. Incycloduction of both eyes was deficient by 2–3 mm during binocular eye movements, while a 1–2 mm deficiency was observed during monocular eye movements (Fig. 1). As medial rectus muscle strength was considered acceptable, surgery was proposed for the four horizontal recti muscles.

**Fig. 2:**
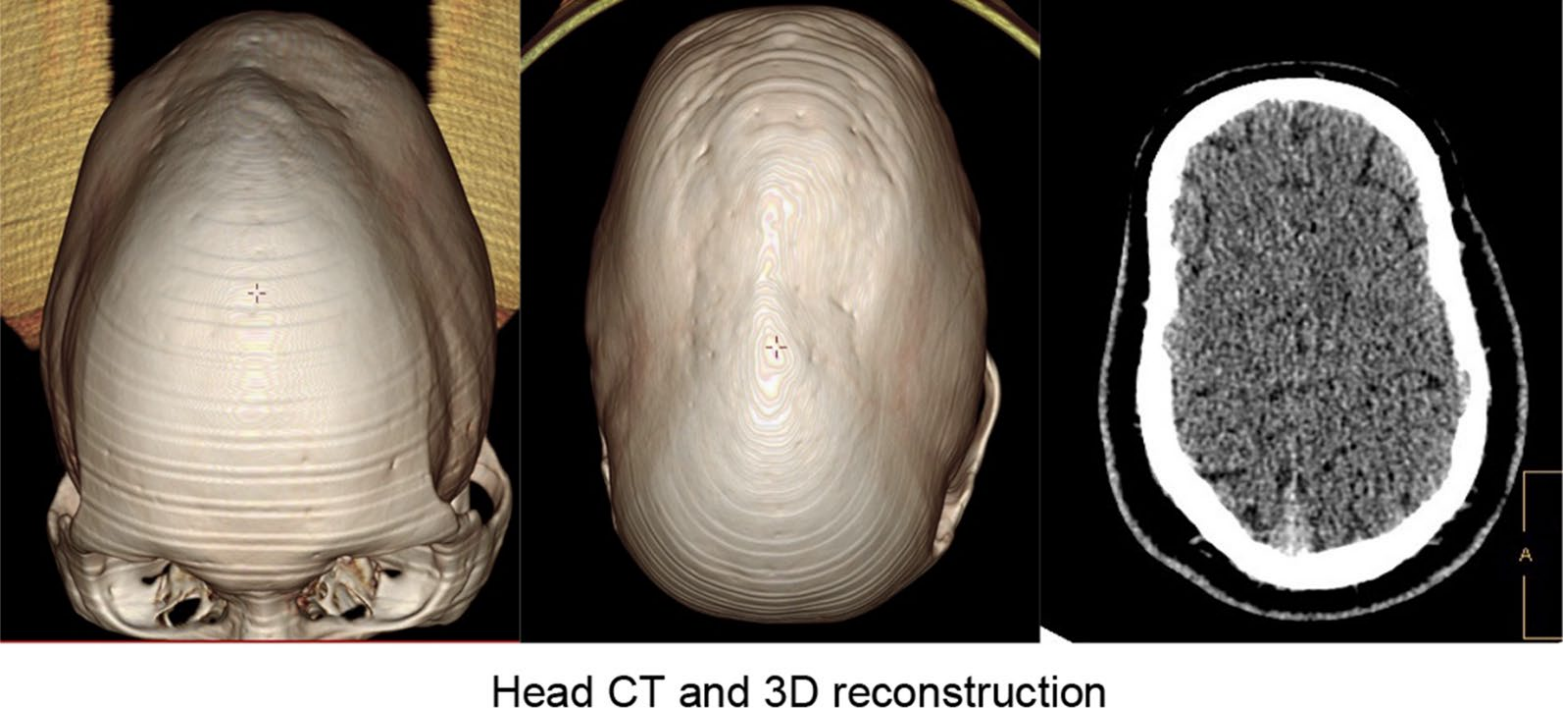
CT scans of the head and a three-dimensional reconstruction of head showing the narrow and elongated shape of the patient’s cranium. The superior region of the forehead appeared pointed and there was a partial upward projection of the skull. The cranial sutures were fused and the overall shape of the head resembled that of a boat

**Fig. 3:**
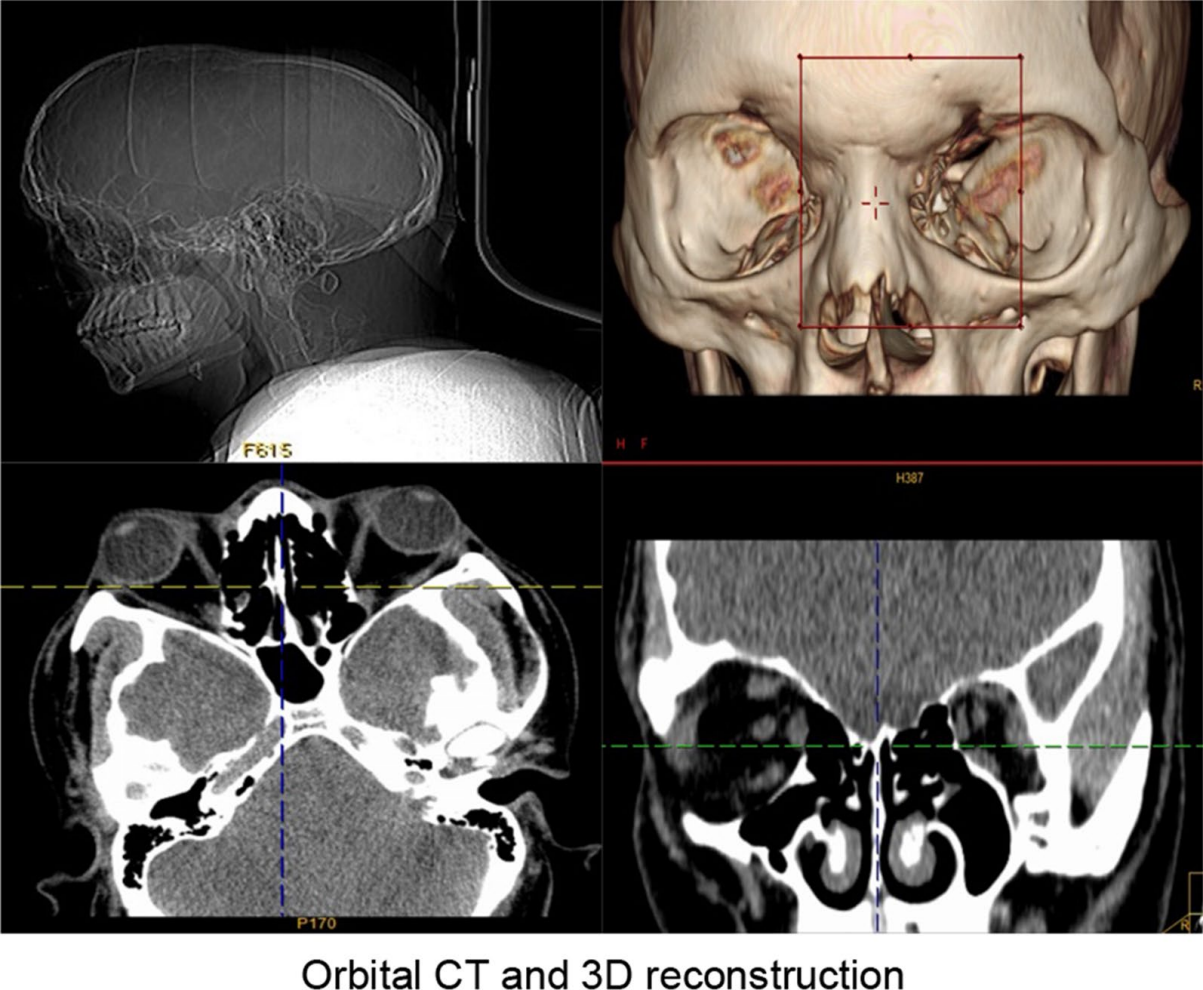
CT scans of the orbits and a three-dimensional reconstruction of the orbits showing shallowed orbits, prominent eyeballs, and slight medial shifts in recti muscles within both eyes

**Fig. 4:**
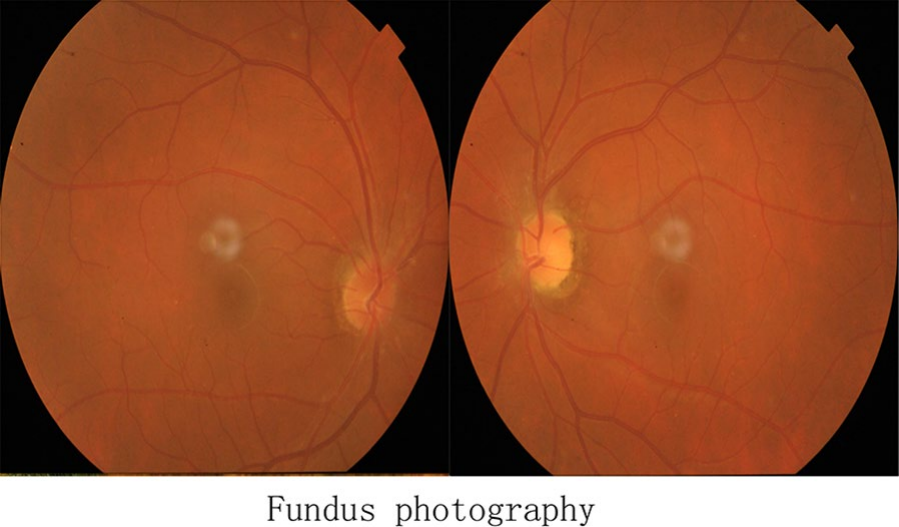
Fundus photograph of both eyes showing no obvious abnormal findings

Results of the computed tomography (CT) scan of the head (Fig. 2) confirmed that the head was narrow and elongated, with the top of the forehead being pointed and a carina projecting partially upward. Cranial sutures were fused and the overall shape of the head resembled that of a boat. CT scan of the orbit (Fig. 3) showed that the orbits were shallow with both eyeballs showing exophthalmos. Fundus photograph of both eyes (Fig. 4) showed no obvious abnormal findings. Peripheral blood samples from the patient, his parents, and younger sisters were extracted for assay of entire exome sequencing DNA testing. Both the patient and his father possessed the *FGFR2* gene c.G812T:p.G271V heterozygous mutation, while the wild-type *FGFR2* gene was present in the patient’s mother and sisters (Fig. 6).

**Fig. 5:**
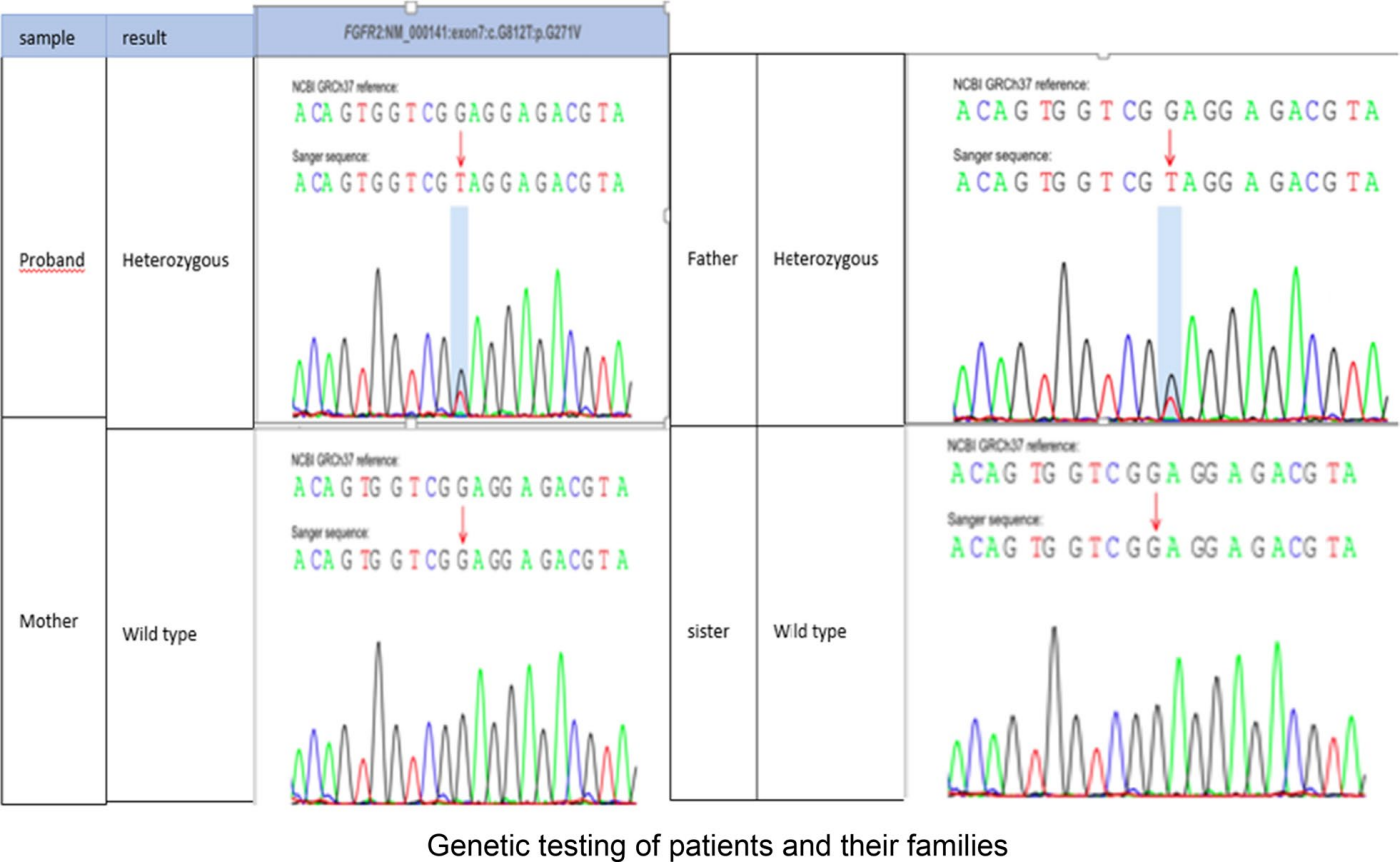
DNA test results showing that both the patient and his father possessed the *FGFR2* gene c.G812T:p.G271V heterozygous mutation, while the wild-type *FGFR2* gene was present in his mother and sisters

**Fig. 6:**
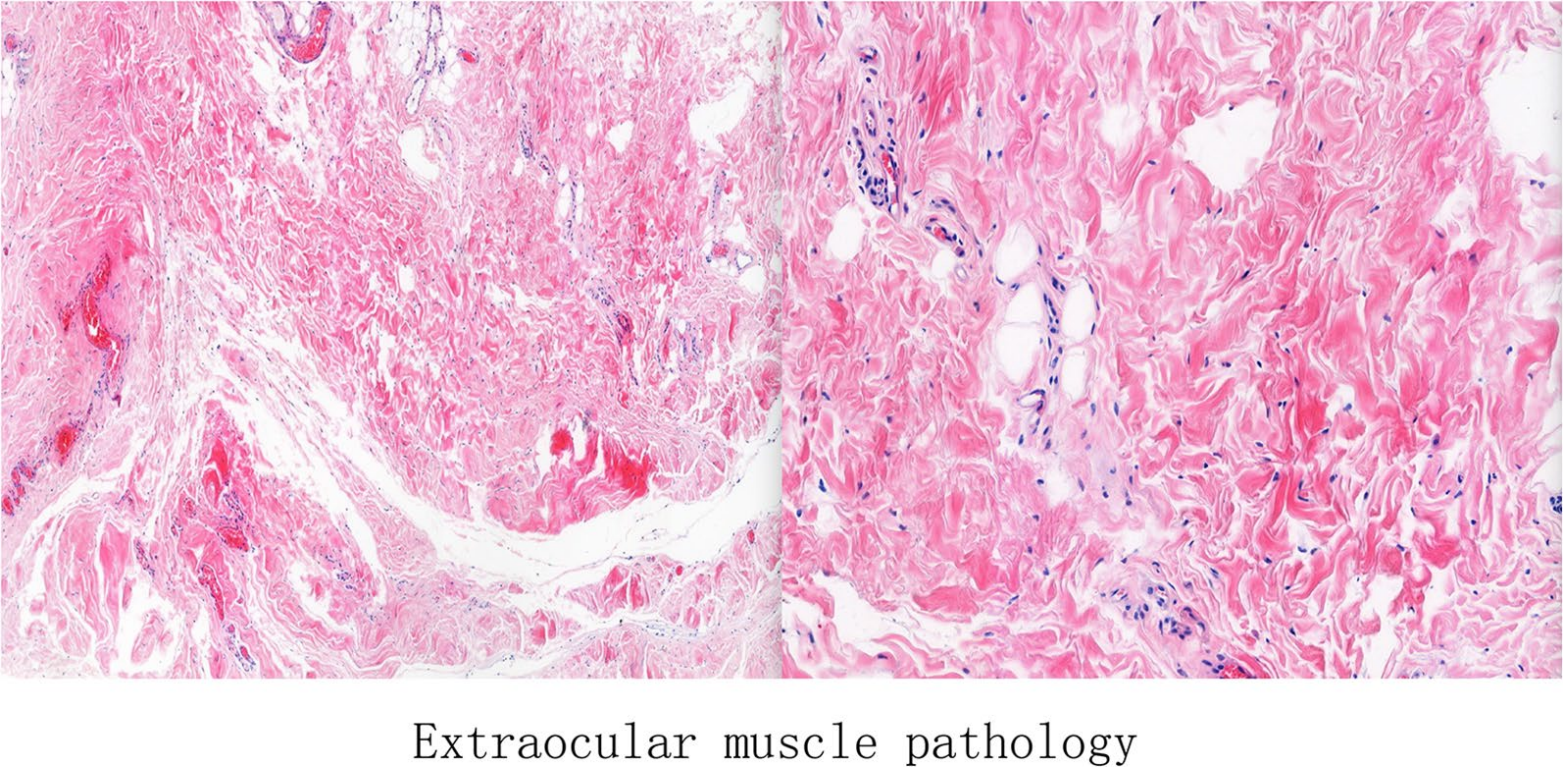
Pathological examination of extraocular muscles showing degeneration of striated muscle fibers, which were replaced with adipose tissue

**Fig. 7:**
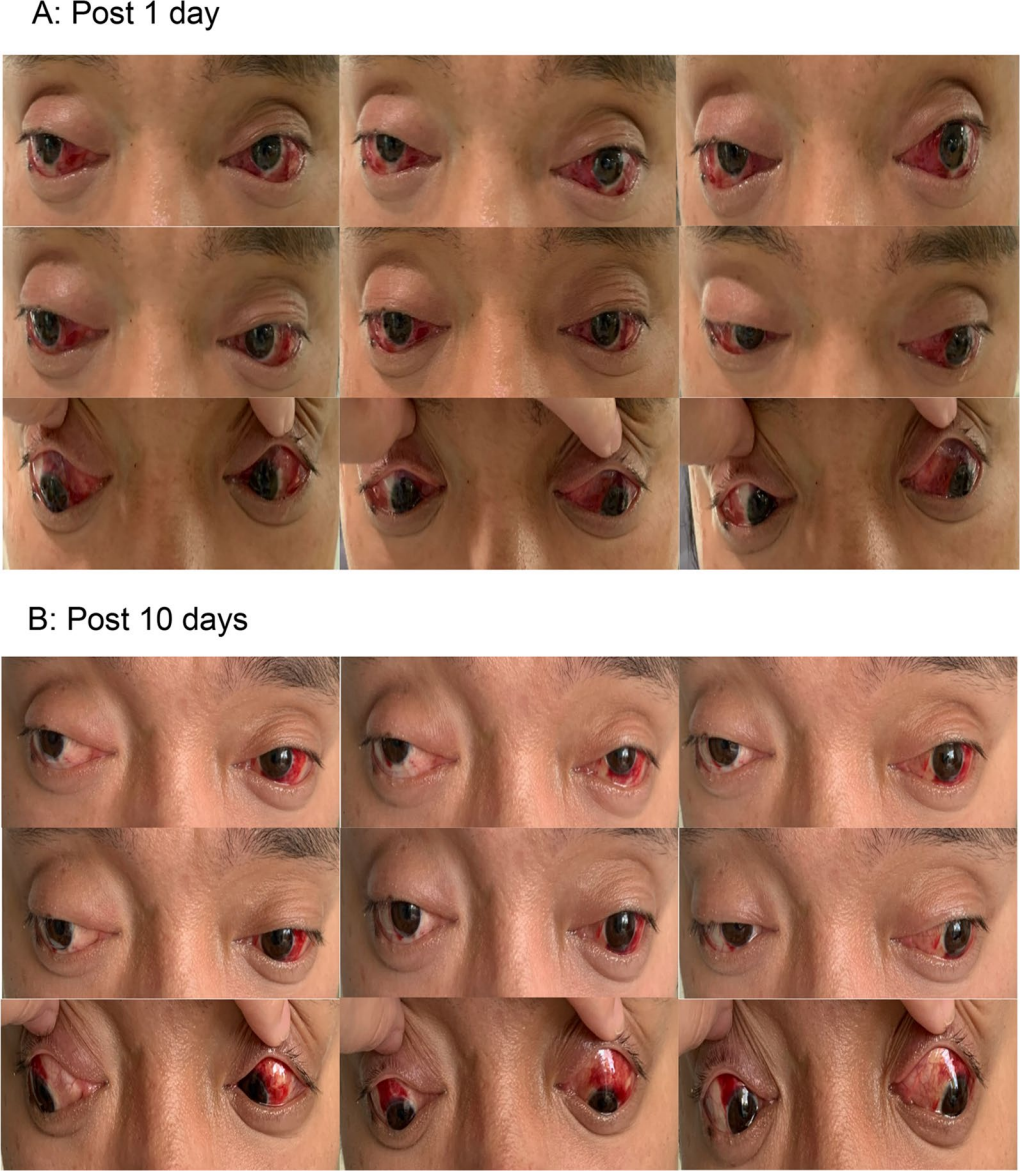
Eye positions in nine directions of gaze as assessed at days 1 (**A**) and 10 (**B**) after surgery. Normal eye positions were present and internal rotations of both eyes were significantly improved

When assessed while the patient was under local anesthesia, lateral rectus muscles in both eyes were recessed 6 mm posteriorly and suspended 3 mm superiorly. Medial rectus muscles in both eyes were resectioned by 6 mm. Results of the intraoperative traction test revealed that mild tension was present in lateral rectus muscles. Pathological examination of an extracted portion of the lateral rectus muscle showed degeneration of striated muscle fibers, which were replaced by adipose tissue (Fig. 6). When assessed at days 1 and 10 after surgery, the patient showed orthophoria (Fig. 7). Coordinated eye movements were present in all directions.

This case demonstrates that Crouzon syndrome patients can show a fibroblast growth factor receptor 2 (*FGFR2*) gene c.G812T:p.G271V mutation and display clinical symptoms such as extraocular muscle fibrosis, exotropia, exophthalmos, and a pointed head deformity.

### Timeline

A 27 year-old male patient arrived at our hospital in March 2021 with a bilateral deviation in eye position that had been present since birth. The patient was hospitalized on 24 March 2021 and surgical treatment of his strabismus was performed that day. The patient was discharged on 26 March 2021.

### Diagnostic assessment

A complete systemic and ophthalmological examination was conducted prior to surgery. The patient exhibited no contraindications in response to this surgery. The preoperative assessment consisted of operation risk and antibiotic eye drops and general examination tests performed included thyroid function, routine blood assay, DNA determination, pathological examination, CT scan, and an evaluation of the strabismus. Results of the Hirschberg test indicated that the corneal reflex was ≥ 45°. The prism alternate cover test (PACT) revealed a constant exotropia (XT) of 100∆ at near and distance fixation, 100∆ XT at upward gaze, and 90∆ XT at downward gaze.

### Therapeutic intervention

The patient experienced a large angle exotropia, while the main purpose for his visit was to improve his appearance. We chose a surgical treatment approach and explained the risk and prognosis to the patient, such as the potential for reoccurrence of exotropia and exacerbation of exophthalmos.

### Follow-up and outcomes

Eye positions in nine directions of gaze were performed at days 1 and 10 after surgery, with the results that normal eye positions were present and internal rotations of both eyes were significantly improved. The patient expressed satisfaction with these postoperative results.

## Discussion

Crouzon syndrome is a rare genetic disorder that manifests in a variety of abnormalities. This condition results from cranial dysplasia, mainly due to a premature closure of coronal sutures and an absence of sutures [12, 13]. The notable abnormalities associated with Crouzon syndrome include a pointed head, parrot-like nose, exophthalmos, shallow orbital cavities, exotropia, optic nerve atrophy, nystagmus, and upper/lower crossbites of the teeth. Treatment is based on the severity of dysfunction and need for cosmetic restoration. In this case, normal vision, fundus and mental development were present. The main symptoms in this patient consisted of a large-angle exotropia and exophthalmos, and therefore corrective strabismus surgery was performed.

The diagnosis of this patient required that it be differentiated from other diseases. As normal thyroid function was present, this excluded thyroid-related eye diseases. Other considerations included Pfeiffer syndrome, mid-face hypoplasia, eye deformities, and deformities of the thumb or toe. It has been reported that Crouzon syndrome can be accompanied with severe hand and foot deformities [12], which also represent classic characteristics of Pfeiffer syndrome. Interestingly, genotype–phenotype studies of Crouzon and Pfeiffer syndrome have shown that a closely overlapping *FGFR2* mutation spectrum exists between the two, indicating that these two conditions may be on a continuum of disorders [6]. The relationship between Crouzon and Pfeiffer syndromes, as related to gene mutation sites, requires further, detailed investigations. As the hands and feet in this case showed normal development, Pfeiffer syndrome was excluded. In our case, DNA testing revealed that this patient possessed a *FGFR2* gene c.G812T:p.G271V heterozygous mutation.

It has been reported that 30–60% of Crouzon syndrome patients involve sporadic cases, which manifest as new mutations [14, 15]. It has been well established that many different mutations in the *FGFR2* gene are associated with Crouzon syndrome. As based on molecular genetic analysis, Glaser *et al*. [16] demonstrated that mutations in some sporadic cases are of paternal origin. In specific, it is believed that Crouzon syndrome may be related with paternal age, with the older the father, the more likely the germline mutation. However, Goriely *et al*. [17] reported that some sporadic cases of the chimera of this gene mutation were of maternal origin. Crouzon syndrome is considered as a very unique single-gene defect disease, primarily involving *FGFR2* [16]. In general, common gene defects are manifest in different clinical phenotypes and can present as different syndromes [18].

In cases of Crouzon syndrome with strabismus, as reported by Coats *et al*., an absence of extraocular muscles are often observed. The orbital CT of the patient in our report did possess extraocular muscles, however, these extraocular muscles were fibrotic and the degenerated striated muscle fibers were replaced with adipose tissue. Extraocular muscle fibrosis is an autosomal dominant genetic disease, which has characteristics of a congenital onset and a positive family history. Our current case demonstrates that Crouzon syndrome patients can carry the *FGFR2* gene c.G812T:p.G271V mutation, extraocular muscle fibrosis, and large-angle exotropia. However, the phenotype of this case differs from that of other studies, which have reported phenotypes associated with mutations like c.1012G > C p.G338R and c.866A > C (Gln289Pro) [19–22]. In this case, the proband was found to have a new c.G812T:p.G271V heterozygous mutation in the *FGFR2* gene. With this mutation, the proband and his father here manifested with the phenotype of lateral rectus muscle fiber degeneration, exotropia, exophthalmos, and a pointed head deformity. However, as reported in other studies, patients with other mutations in the *FGFR2* gene manifested with other phenotypes: patients with the c.1012G > C p.G338R mutation manifested with the phenotype of ocular proptosis, shallow orbits, and mid-face hypoplasia; patients with the mutation c.866A > C (Gln289Pro) in this gene manifested with the phenotype of shallow orbits and ocular proptosis, accompanied by mid-face hypoplasia, craniosynostosis, and clinically normal hands and feet. Accordingly, the presence of extraocular muscle fibrosis, as related to the gene mutation in Crouzon syndrome, will require further study and additional cases.

## Conclusions

Our case demonstrates that patients with Crouzon syndrome can show an *FGFR2* gene c.G812T:p.G271V mutation along with clinical symptoms consisting of lateral rectus muscle fiber degeneration, exotropia, exophthalmos, and a pointed head deformity. To the best of our knowledge, this case represents the first report indicating a link between this *FGFR2* gene mutation and Crouzon syndrome. In addition, in this adult patient with Crouzon syndrome, normal mental, facial, and extraocular muscle development was present.

## Data Availability

Not applicable.
